# Interactions between cortisol and lipids in human milk

**DOI:** 10.1186/s13006-020-00307-7

**Published:** 2020-07-20

**Authors:** Kaisa M. Linderborg, Maaria Kortesniemi, Anna-Katariina Aatsinki, Linnea Karlsson, Hasse Karlsson, Baoru Yang, Henna-Maria Uusitupa

**Affiliations:** 1 Department of Biochemistry, Food Chemistry and Food Development, University of Turku, Itäinen Pitkäkatu 4C, FI-20014 Turun yliopisto, Turku, Finland; 2 The FinnBrain Birth Cohort Study, Turku Brain and Mind Center, Institute of Clinical Medicine, University of Turku, Turku, Finland; 3 Department of Child Psychiatry, Turku University Hospital and University of Turku, Turku, Finland; 4 Department of Psychiatry, Turku University Hospital and University of Turku, Turku, Finland

**Keywords:** Cortisol, Fatty acids, Human milk, Lipids, Phospholipids, Triacylglycerols

## Abstract

**Background:**

Human breast milk is one of the key early postnatal biological exposures for the developing child. It includes bioactive compounds, such as cortisol and fatty acids, which may be linked via the mother’s lipid metabolism.

**Methods:**

This study investigated the associations between cortisol and lipids in human milk at the infant age of 2.5 months. Human milk cortisol concentrations were measured using luminescence immunoassay, and two groups of milks (*n* = 50 each) were formed based on either high (> 10 nmol/L) or low (< 3 nmol/L) cortisol levels. Lipids, as fatty acid content and composition of neutral (triacylglycerol-rich) and polar (phospholipid-rich) lipids, were measured with gas chromatography. The samples originated from the FinnBrain Birth Cohort Study.

**Results:**

The percentage of phospholipid-rich lipids of total lipids was 33.08% ± 1.33%. In triacylglycerol-rich lipids, high cortisol level in milk was associated with higher lauric (12:0, mass % and mg/mL), myristic (14:0, mass % and mg/mL), eicosenoic (20:1*n* − 9, mass %), docosenoic (22:1*n* − 9, mass %, and mg/mL) acids, and to lower palmitic acid (16:0, mass %) compared with low cortisol levels in milk. In phospholipid-rich lipids, high cortisol level was associated with higher myristic (14:0, mass %) and docosenoic (22:1*n* − 9, mass %) acids. After adjusting for pre-pregnancy BMI and sampling time by linear regression, the milk cortisol remained a significant predictor for lauric and myristic acids in triacylglycerol-rich lipids, and myristic and docosenoic acid in phospholipid-rich lipids (β = 0.23 to 0.38 and *p* < 0.05 for each).

**Conclusions:**

This study revealed certain significant associations between milk cortisol and the fatty acid composition of human milk, indicating that cortisol might be one of the factors affecting the origin of the lipids in human milk.

## Background

### Plasma and milk cortisol and cortisol’s effects on lipid metabolism

Human breast milk contains a number of bioactive compounds, and thus it serves as one of the key early postnatal biological exposures for the developing child. Bioactive constituents in human milk include cortisol, fatty acids (FAs), such as polyunsaturated fatty acids (PUFAs), and parts of the milk fat globule membrane. These are all linked, as the metabolic and endocrine activities of adipose tissue, and energy metabolism in general, are affected by glucocorticoids, cortisol and cortisone [1]. Both cortisol and cortisone are secreted from the cortex of the adrenal glands in response to physiological and psychological stress [2]. Of these, especially cortisol plays a key role in gluconeogenesis, lipolysis and energy metabolism and thus potentially affects the lipid content and composition of human milk. Acutely, cortisol may play a role in the utilization of different lipid sources for the production of the lipids of human milk [3–6], while chronic exposure to cortisol has been linked with lipogenesis and obesity [7]. The linkage between lipids and cortisol may be mediated via cytokines. Adipose tissue can secrete pro-inflammatory cytokines and stimulate the hypothalamic-pituitary-adrenal axis, and cortisol decreases the production of cytokines and other inflammatory mediators [8, 9]. The use of antenatal corticosteroids may increase the total lipid content in human milk following premature birth (< 34 gestational weeks) [10], which further substantiates the need for investigations into the relationship between milk cortisol and milk lipids. Milk cortisol, on the other hand, has been shown to significantly correlate with the mother’s plasma cortisol [11]. The role of milk glucocorticoids in infant development is not fully understood as yet, but it has been suggested that they are involved in neural development, intestinal maturation, development of microbiota and programming the infant metabolism [12, 13] as well as temperament traits that describe individual differences in emotional reactivity and self-regulation [14]. It has also been indicated that glucocorticoid and bile acid metabolism are linked, and that glucocorticoids play a role in the development of diseases related with obesity such as liver diseases and hypertension [7, 15]. While the composition of human milk is previously known to be influenced by gestational age [10], geographical origin [16, 17], dietary habits [10, 18–21], maternal body mass index (BMI) [20], socio-economic situation [22] and even infant gender [23], there is scarce knowledge about the associations between cortisol and the lipid composition in human milk. However, milk cortisol has been shown to positively correlate with the fat content of the milk of rhesus monkeys (*Macaca mulatta*) [24], but in humans no association between the total fat content in milk and milk cortisol has been established [25].

### Origin of milk lipids and their importance for the infant

Lipids in breast milk or infant formula are the major sources of energy and the sole supply of essential FAs to the infant during the first months of its life. Breast milk contains a complex mixture of different lipids with a quantitative dominance of triacylglycerols (TAGs). The FAs used by the mammary gland for the synthesis of TAGs originate from the mother’s diet, mother’s FA stores, or from the mother’s de novo lipid synthesis. The milk-fat globule membrane, consisting mainly of phospholipids (PLs), pinches off from the mother’s cell membrane [26]. The lipid content and composition in the mother’s diet as well as the mother’s dietary pattern influence the utilization of FAs for TAG synthesis by the mammary epithelial cells [27]. De novo lipogenesis is the endogenous synthesis of FAs. Increased production of TAGs by de novo lipogenesis increases the prevalence of medium-chain FAs in milk. Thus, medium-chain FAs are more abundant in the milk produced by mothers who eat a very low fat but adequate calorie diet compared to mothers on a diet containing about 40% of calories as fat [15]. Existing lipid stores are utilized especially during the mother’s fasting state or in the absence of dietary fat [27]. Humans can synthesize saturated and monounsaturated FAs, while the essential FAs, linoleic (18:2*n* − 6) and α-linolenic (18:3*n* − 3) acids, must originate from the diet. PUFAs, such as arachidonic (20:4*n* − 6), eicosapentaenoic (20:5*n* − 3) and docosahexaenoic (22:6*n* − 3) acids, originate from the elongation and desaturation of the essential FAs or directly from diet, most importantly from marine sources. Although the crucial need of bioactive long-chain PUFAs for the normal development of the infant is scientifically established [28–30], the optimal composition of breast milk lipids remains unknown.

In enterocytes, lipids originating from the diet are packaged into chylomicrons, which are further secreted into blood via the lymphatics to deliver their fat to mammary glands during lactation. In addition to chylomicrons, very low-density lipoproteins originating from the liver carry abundant quantities of TAGs. In order for the TAGs of chylomicrons or very low-density lipoproteins to be transported from the circulation to the milk, they must be hydrolyzed by lipoprotein lipase. Mammary adipocytes have been suggested to be the source of lipoprotein lipase in the mammary gland [31], possibly further explaining associations between the mother’s lipid metabolism and the composition of human milk.

The date of the last literature search on the topic was January 4^th^, 2020 by using the key words breast milk, cortisol, fatty acids, human milk, lipids, phospholipids and triacylglycerols.

### Aims of the study

To better understand the factors affecting the composition of human milk, we investigated the associations of milk cortisol with the FA content and the composition in two different lipid classes, the TAG- and PL-rich lipids of human milk.

## Methods

### Participants and breast milk collection

**Fig. 1:**
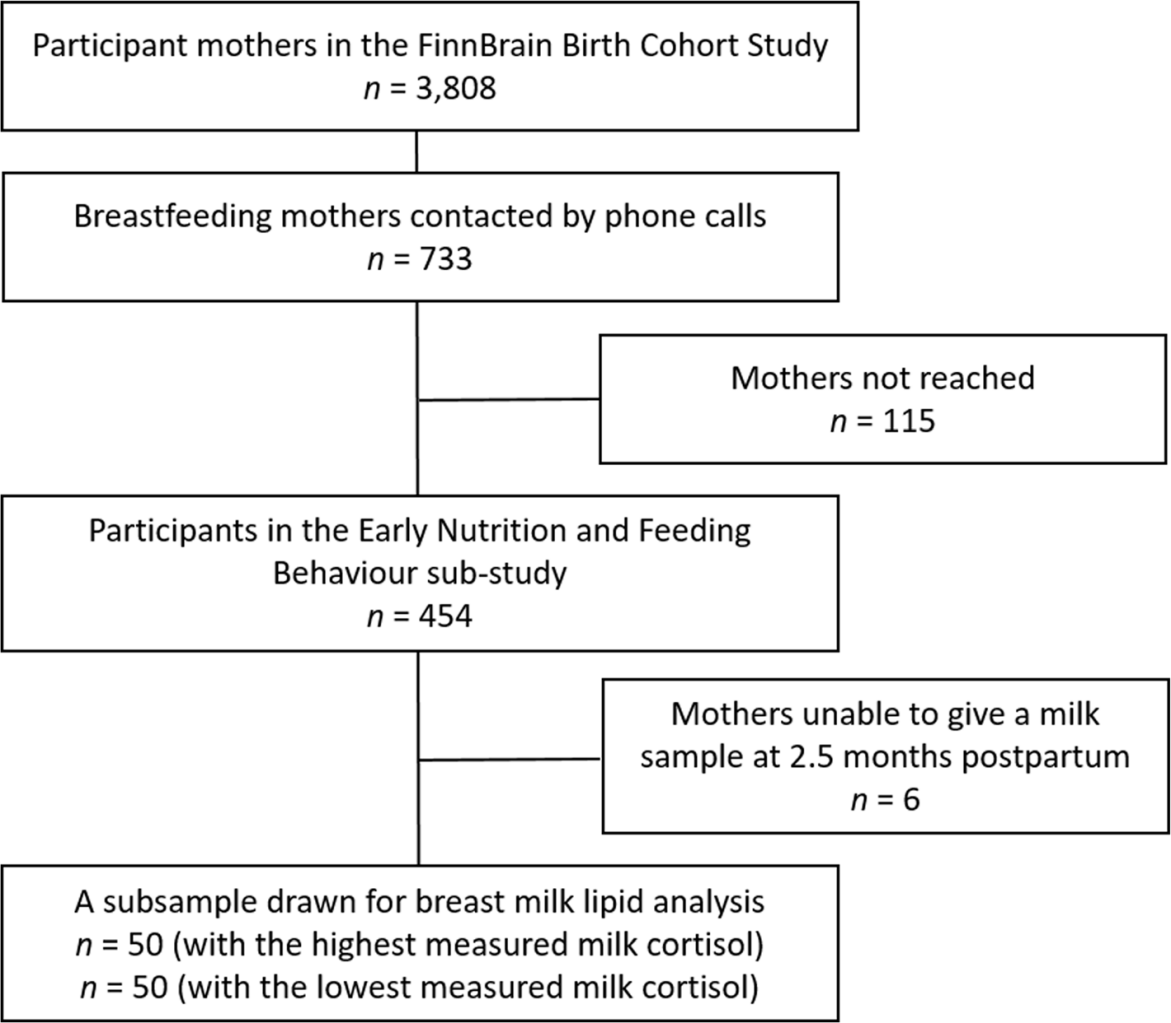
Flow chart of the study participants and design

FinnBrain Birth Cohort Study is an on-going, intergenerational prospective observational study conducted at the University of Turku, Finland. Mothers were recruited to the FinnBrain Birth Cohort Study [32] during their first trimester ultrasound visit by a research nurse. Of the baseline characteristics, parity, education level (grouped as “Mid/Low” [levels 1–5 in the Finnish education system, secondary school/vocational education or lower], “High/Voc” [level 6: polytechnic education], “High” [levels 7–9: university/graduate school]) and exclusivity of breastfeeding were self-reported. Diagnosis of gestational diabetes, pre-pregnancy BMI, birth mode, gestational age at birth, child’s birth weight, sex and mother’s age were obtained from the Finnish Medical Birth Register, maintained by the Finnish National Institute for Health and Welfare, Finland. Medication was not considered as an exclusion criteria. From the larger FinnBrain Birth Cohort Study [32], Finnish mothers who breastfed their infants were recruited by phone calls to the early nutrition and feeding behavior sub-study (Fig. 1). A total of 733 mothers were contacted and 618 reached. Of these, 454 agreed to participate in the sub-study, and 448 gave a breast milk sample at the infant age of 2.5 months. The milk samples were self-collected by mothers in the presence of a study nurse during a study visit in the research facility. The 100 samples were collected as follows: Year: 50 samples were collected in 2014, 49 in 2015 and one in 2016. For the time of the year: 22 samples were collected in winter (January to February), 33 in spring (March to May), 21 in summer (June to August) and 24 in autumn (September to December). Weekday: on Monday, 4 samples were collected; on Tuesday, 21 were collected; on Wednesday, 33 were collected; on Thursday, 16 were collected; and on Friday, 26 were collected. Visits during the afternoon were encouraged to minimize the effect of diurnal variation in cortisol. Sampling time is presented as minutes after 8:00 am in Table 1, and it was used as a covariate in statistical analyses. The mothers were instructed to feed the baby from the right breast 1.5–2 h prior to the study visit and thereafter refrain from feeding from the right breast before taking the study sample. Breastfeeding from the left breast was not restricted. After entering the research facility mothers were asked to wear latex gloves and express 10 mL of front milk with manual expression from the right breast. The samples were immediately transferred to and kept in a − 70 °C freezer until analyzed. For the present study, two subpopulations of fifty mothers each were drawn from the population, who had given breast milk samples based on the milk previously determined [14] milk cortisol levels (< 3 nmol/L, mean ± SD = 2.02 +/− 0.44 or > 10 nmol/L, mean ± SD = 15.2 ± 5.89 nmol/L) in order to investigate differences in lipid content based on milk cortisol groups. The baseline characteristics of the dyads are presented in Table 1.

**Table 1:** Characteristics of the mother-infant dyads by low and high milk cortisol groups

	Whole population (*n* = 100)	Low milk cortisol < 3 nmol/L (*n* = 50)	High milk cortisol > 10 nmol/L (n = 50)	T-test^a^ / *χ*^2^	OR (95% CI)^b^
Gestational age	40 [36–42]	40 [36–42]	40 [37–42]	t = − 1.49 (*p* = 0.14)	
Infant gender				*χ*^2^ = 0.16 (*p* = 0.67)	1.28 (0.58, 2.81)
Boy	55 (55%)	29 (58%)	26 (52%)		
Girl	45 (45%)	21 (42%)	24 (48%)		
Birth mode				*χ*^2^ = 0.08 (*p* = 0.77)	0.72 (0.23, 2.24)
Vaginal delivery	86 (86%)	44 (88%)	42 (84%)		
Cesarean section	14 (14%)	6 (12%)	8 (16%)		
Mother’s BMI	24 [18–39]	25 [19–39]	24 [18–37]	t = 1.74 (*p* = 0.09)	
Mother’s age	30 [24–44]	31 [24–44]	30 [24–37]	t = 2.24 (p = 0.03)	
Education level				*χ*^2^ = 1.26 (*p* = 0.53)^c^	
Secondary school / vocational education or lower	22 (22%)	12 (24%)	10 (20%)		
Polytechnic	38 (38%)	17 (34%)	21 (42%)		
University/graduate school	33 (33%)	19 (38%)	14 (28%)		
Missing data	7 (7%)	2 (4%)	5 (10%)		
Primiparous				*χ*^2^ = 2.69 (*p* = 0.10)	2.15 (0.95, 4.90)
No	39 (39%)	24 (48%)	15 (30%)		
Yes	61 (61%)	26 (52%)	35 (70%)		
Gestational diabetes				*χ*^2^ = 2.82 (p = 0.09)	3.24 (0.96, 11.00)
Yes	15 (15%)	11 (22%)	4 (8%)		
No	85 (85%)	39 (78%)	46 (92%)		
Exclusive breastfeeding				*χ*^2^ = 0.9 (*p* = 0.34)	0.48 (0.15, 1.56)
No	14 (14%)	5 (10%)	9 (18%)		
Yes	84 (84%)	45 (90%)	39 (78%)		
Missing data	2 (2%)	0 (0%)	2 (4%)		
Birth weight	3656 [2705–4940]	3622 [2830–4940]	3690 [2705–4660]	t = −0.69 (*p* = 0.49)	
Milk cortisol concentration (nmol/L)	9 [1–34]	2 [1–3]	15 [10–34]	t = − 15.81 (*p* < 0.001)	
Sampling time as minutes after 08:00 am	319 [30–645]	354 [120–645]	284 [30–555]	t = 2.93 (*p* = 0.004)^d^	
Infant age at sampling (days)	61 [41–106]	60 [41–85]	62 [46–106]	t = − 0.94 (*p* = 0.35)	

Data is given as mean or number with either range in square brackets or percentage in parentheses

^a^ df = 98

^b^ Crude odds ratio with 95% confidence interval

^c^ χ^2^ for trend: Linear-to-linear association 0.13, df = 1, *p* = 0.719

^d^ Data available from 47 dyads in the low cortisol group and 46 dyads in the high cortisol group (df = 91)

### Milk cortisol

Milk was thawed at room temperature and gently mixed for 1 min prior to dichloromethane extraction. Cortisol was analyzed using a validated luminescence immunoassay method (IBL International, product RE62111) [33] at the Finnish Institute of Occupational Health. Values are expressed as nmol/L.

### Fatty acid content and composition of triacylglycerol-rich and phospholipid-rich fractions of milk

The FA compositions of neutral lipid (i.e., the TAG-rich fraction) and polar lipid (i.e., the PL-rich fraction) fractions were analyzed from each sample in duplicate. Samples were thawed at room temperature and gently mixed, after which the internal standard mixture consisting of triheptadecanoin (Larodan Fine Chemicals AB, Malmö, Sweden) and dinonadecanoylphosphatidylcholine (Larodan Fine Chemicals AB, Malmö, Sweden) was added to the sample. The total lipids were extracted from samples with a modified Folch procedure [34] with chloroform, methanol and 0.88% potassium chloride in water in a two-phase extraction to maximize the yield. Extracted lipids were further separated into TAG-rich and PL-rich fractions by solid phase chromatography using silica cartridges (Waters, Dublin, Ireland) [35]. The TAG-rich fraction was eluted from the column with high-performance liquid chromatography grade dry diethyl ether (Merck KGaA, Germany) and the PL-rich fraction with high performance liquid chromatography grade methanol (Honeywell, Riedel-de Haen, France). Fatty acid methyl esters were prepared from the isolated fractions with sodium-methoxide-catalyzed transesterification. The methyl esters were analyzed with Shimadzu GC-2010 with an AOC-20i auto injector and flame ionization detector and GCsolution software (Shimadzu Corp., Kyoto, Japan). Splitless/split injection with a split opened after 1 min was used. A wall coated, open tubular column DB-23 (60 m × 0.25 mm i.d., liquid film 0.25 μm, Agilent Technologies, J.W. Scientific, Santa Clara, CA, USA) was used to separate the methyl esters. The following settings were used: injector at 270 °C, oven initial at 130 °C, hold 1 min, rate 4.5 °C/min to 170 °C, hold 0 min, rate 10 °C/min to 220 °C, hold 14.5 min, rate 60 °C/min to 230 °C, hold 3 min and detector at 280 °C. Helium was used as carrier gas and 68D (Nu-Check-Prep, Elysian, MN, USA), Supelco 37 Component Fatty Acid Methyl Ester Mix (Supelco, St. Louis, MO, USA), and 11A (Nu-Check-Prep, Elysian, MN, USA) as external standards. Results were expressed as molar percentage of each FA in its lipid category and as mg in mL milk. Additionally, the percentage of polar lipids (as phosphatidylcholines) from all lipids was calculated. The quantity (mg/mL) is presented as indication of the absolute amount of FAs received by the infant. However, human milk is known to vary in its lipid content even within the same feeding. Thus, relative proportions (percentages of certain FAs of all FAs), which are an indication of the FA balance within the different FA families (SAFAs, MUFAs, omega-3 PUFAs and omega-6 PUFAs), are also presented. Fatty acids were named both with their common names as well as the systematic numeric abbreviation. The latter represents the number of carbons in the acyl chain followed by the number of double bonds in the acyl chain as well as the location of the first double bond from the omega end of the acyl chain, for example oleic acid (18:1*n* − 9). Quantification was based on internal standards. Odd numbered FAs were chosen as internal standards, as their chromatographic behavior is similar to that of even numbered FAs, but they are not expected to be present in the milk lipids in substantiated amounts.

### Statistical methods

Statistical analyses were performed with the SPSS version 23 program (IBM, Armonk, NY, USA). Multivariate models were performed by using Unscrambler X, version 10.4 (CAMO Software, Oslo, Norway). All data were checked for normality and homogeneity of variance and reported as means ± standard deviation. Statistical significance was determined at *p* < 0.05. Independent samples T-test or Mann–Whitney *U* test, depending on the normality of the data, was used to assess significant differences between the high and low cortisol groups. A Pearson Chi-Square and odds ratios were used for categorized variables. Principal component analysis (PCA) was applied for standardized data to study the differences and possible classifications among samples. Cross validation was used to estimate the number of components for a statistically reliable model. Linear regression models were built for the lipids to adjust milk cortisol for the pre-pregnancy BMI and milk sampling time. For these models, milk cortisol (as a continuous variable) was transformed to its natural logarithm, and missing values for sampling time (*n* = 7) were imputed with mean value of the other cases.

## Results

### Characteristics of the mother-infant dyads

There were no differences in the gestational age, birth weight, infant age at the milk sampling time, infant gender, vaginal delivery, education level, parity, or exclusivity of breastfeeding between the groups formed based on milk cortisol levels (Table 1). Low milk cortisol was associated with higher pre-pregnancy BMI when compared with high milk cortisol, and there was a significant difference in the sampling time measured as minutes after 8:00 am between the low and high cortisol groups (t = 2.93, df = 91, *p* = 0.004; mean ± SD = 354 ± 119 vs. 284 ± 108 min, respectively; Table 1). Only two of the mothers reported themselves as smokers.

### Fatty acid content and composition of triacylglycerol-rich and phospholipid-rich fractions

Results expressed as mg/mL deviated more from sample to sample than relative proportions (% of all FAs in TAG-rich and PL-rich fractions) due to the expected variation [26] in the lipid content of human milk (Table 2, Table 3). The content of TAG-rich or PL-rich lipids did not differ between the groups formed.

**Table 2:** Fatty acid compositions of triacylglycerol-rich lipids in human milk

Fatty acid	Relative mass proportions in triacylglycerol-rich lipids					Quantities as triacylglycerols (mg/mL)				
	Low cortisol (< 3 nmol/L)		High cortisol (> 10 nmol/L)		*p*-value	Low cortisol (< 3 nmol/L)		High cortisol (> 10 nmol/L)		p-value
	Mean	STDEV	Mean	STDEV		Mean	STDEV	Mean	STDEV	
Lauric 12:0	3.29	1.22	4.28	1.50	**0.001**^a,b^	1.07	0.61	1.53	0.89	**0.005**^b,c^
Myristic 14:0	5.37	1.36	6.09	1.53	**0.02**^a,b^	1.70	0.82	2.12	0.96	**0.02**^b,c^
Myristoleic 14:1*n* − 5	0.28	0.08	0.26	0.08	0.25^a^	0.09	0.05	0.09	0.04	0.86^c^
Palmitic 16:0	23.10	2.55	22.28	2.35	**0.03**^c^	7.26	2.98	7.74	2.90	0.41^c^
Palmitoleic 16:1*n* − 7	2.77	0.68	2.59	0.78	0.11^c^	0.89	0.45	0.91	0.46	0.82^c^
Stearic 18:0	7.16	1.57	6.93	1.48	0.88^c^	2.22	0.93	2.39	0.98	0.53^c^
Oleic 18:1*n* − 9	39.20	3.32	38.92	3.28	0.71^c^	12.35	4.83	13.74	5.95	0.51^c^
Vaccenic 18:1*n* − 7	1.99	0.25	1.99	0.24	0.70^c^	0.64	0.27	0.70	0.30	0.49^c^
Linoleic 18:2*n* − 6	10.62	1.99	10.46	2.10	0.52^c^	3.32	1.32	3.73	1.86	0.59^c^
Linolenic 18:3*n* − 3	1.73	0.56	1.84	0.57	0.48^c^	0.54	0.26	0.65	0.36	0.18^c^
Arachidic 20:0	0.19	0.06	0.19	0.04	0.20^c^	0.06	0.03	0.07	0.02	0.14^c^
Gondoic 20:1*n* − 9	0.46	0.11	0.50	0.11	**0.01**^c^	0.14	0.07	0.18	0.09	0.07^c^
Eicosadienoic 20:2*n* − 6	0.25	0.04	0.25	0.05	0.68^a^	0.08	0.03	0.09	0.04	0.30^c^
Dihomogammalinolenic 20:3*n* − 6	0.33	0.07	0.30	0.08	0.14^c^	0.10	0.04	0.11	0.06	0.95^c^
Arachidonic 20:4*n* − 6	0.39	0.09	0.38	0.13	0.17^c^	0.12	0.06	0.14	0.12	0.98^c^
Eicosatrienoic 20:3*n* − 3	0.04	0.04	0.06	0.04	**0.05**^c^	0.02	0.01	0.02	0.02	0.13^c^
Eicosapentaenoic 20:5*n* − 3	0.12	0.08	0.12	0.08	0.26^c^	0.04	0.02	0.04	0.04	0.65^c^
Behenic 22:0	0.03	0.03	0.04	0.03	0.44^c^	0.01	0.01	0.01	0.01	0.44^c^
Docosenoic 22:1*n* − 9	0.07	0.04	0.10	0.03	**0.008**^c^	0.02	0.02	0.03	0.02	**0.03**^c^
Nervonic 24:1*n* − 9	0.37	0.19	0.35	0.24	0.27^c^	0.11	0.07	0.13	0.14	0.80^c^
Docosahexaenoic 22:6*n* − 3	0.01	0.08	0.02	0.05	0.16^c^	0.00	0.02	0.01	0.02	0.16^c^
Other fatty acids	2.23	0.37	2.05	0.45	**0.04**^a^	0.71	0.32	0.71	0.27	0.91^c^
SAFA	39.14	5.05	39.80	4.18	0.57^c^	12.32	5.12	13.87	5.21	0.22^c^
MUFA	44.77	3.46	44.36	3.31	0.55^a^	14.14	5.57	15.65	6.67	0.49^c^
PUFA	13.50	2.47	13.43	2.67	0.65^c^	4.23	1.67	4.79	2.41	0.50^c^
Omega-3 FAs	1.91	0.59	2.03	0.62	0.50^c^	0.60	0.29	0.72	0.41	0.18^c^
Omega-6 FAs	11.59	2.06	11.40	2.19	0.41^c^	3.63	1.42	4.07	2.03	0.61^c^
						Triacylglycerols (mg/mL)				
						31.52	12.13	35.15	13.85	0.39^c^

Data is presented as relative proportions (%) and quantities (mg/mL) and grouped based on milk cortisol levels. Statistically significant differences (p < 0.05) between low and high milk cortisol groups are highlighted with bold type. Abbreviations: STDEV standard deviation, SAFA saturated fatty acids, MUFA monounsaturated fatty acids, PUFA polyunsaturated fatty acids

^a^ Significant difference between the low and high cortisol groups, T-test (df = 98)

^b^ Milk cortisol (as continuous variable) remained a statistically significant predictor after controlling for pre-pregnancy BMI and sampling time in linear regression analysis, see Additional file 1

^c^ Significant difference between the low and high cortisol groups, Mann–Whitney *U* test

**Table 3:** Fatty acid compositions of phospholipid-rich lipids in human milk

Fatty acid	Relative mass proportions in phospholipid-rich lipids					Quantities as phosphatidylcholine (mg/mL)				
	Low cortisol (< 3 nmol/L)		High cortisol (> 10 nmol/L)		p-value	Low cortisol (< 3 nmol/L)		High cortisol (> 10 nmol/L)		p-value
	Mean	STDEV	Mean	STDEV		Mean	STDEV	Mean	STDEV	
Lauric 12:0	2.16	1.04	2.50	1.38	0.29^a^	0.03	0.02	0.03	0.02	0.63^a^
Myristic 14:0	4.01	1.38	4.87	1.77	**0.01**^a,b^	0.05	0.03	0.05	0.03	0.37^a^
Myristoleic 14:1*n* − 5	0.16	0.11	0.16	0.09	0.99^a^	0.002	0.002	0.002	0.001	0.59^a^
Palmitic 16:0	21.55	3.04	21.70	3.25	0.81^c^	0.23	0.14	0.21	0.10	0.96^a^
Palmitoleic 16:1*n* − 7	1.96	0.63	1.88	0.70	0.57^c^	0.02	0.02	0.02	0.01	0.53^a^
Stearic 18:0	13.20	4.14	13.07	3.83	0.81^a^	0.12	0.06	0.12	0.05	0.52^a^
Oleic 18:1*n* − 9	31.30	6.62	30.13	5.98	0.23^a^	0.37	0.29	0.32	0.20	0.61^a^
Vaccenic 18:1*n* − 7	1.68	0.32	1.63	0.34	0.43^c^	0.02	0.02	0.02	0.01	0.58^a^
Linoleic 18:2*n* − 6	14.81	2.68	14.94	2.72	0.77^a^	0.15	0.09	0.15	0.07	0.69^a^
Linolenic 18:3*n* − 3	1.44	0.58	1.49	0.57	0.98^a^	0.02	0.02	0.02	0.01	0.77^a^
Arachidic 20:0	0.18	0.08	0.19	0.07	0.69^a^	0.002	0.001	0.002	0.001	0.81^a^
Gondoic 20:1*n* − 9	0.63	0.16	0.65	0.15	0.37^a^	0.007	0.004	0.006	0.004	0.88^a^
Eicosadienoic 20:2*n* − 6	0.30	0.07	0.33	0.07	0.06^c^	0.003	0.002	0.003	0.002	0.89^a^
Dihomogammalinolenic 20:3*n* − 6	0.85	0.32	0.86	0.33	0.98^a^	0.008	0.004	0.008	0.004	0.52^a^
Arachidonic 20:4*n* − 6	1.83	0.79	1.92	0.99	0.90^a^	0.02	0.01	0.02	0.01	0.70^a^
Eicosatrienoic 20:3*n* − 3	0.02	0.04	0.01	0.04	0.27^a^	0.000	0.001	0.000	0.001	0.22^a^
Eicosapentaenoic 20:5*n* − 3	0.29	0.16	0.26	0.15	0.26^a^	0.003	0.002	0.003	0.002	0.29^a^
Behenic 22:0	0.03	0.04	0.03	0.04	0.93^a^	0.000	0.001	0.000	0.001	0.93^a^
Docosenoic 22:1*n* − 9	0.11	0.08	0.17	0.11	**0.009**^a,b^	0.001	0.001	0.001	0.001	0.11^a^
Nervonic 24:1*n* − 9	1.02	0.69	0.92	0.39	0.93^a^	0.01	0.01	0.01	0.01	0.48^a^
Docosahexaenoic 22:6*n* − 3	0.05	0.33	0.06	0.28	0.69^a^	0.000	0.001	0.000	0.001	0.71^a^
Other fatty acids	2.43	0.81	2.24	0.70	0.11^a^	0.03	0.02	0.02	0.01	0.50^a^
SAFA	41.13	5.01	42.37	5.15	0.23^c^	0.43	0.25	0.41	0.18	0.99^a^
MUFA	35.83	7.17	34.62	6.50	0.20^a^	0.43	0.33	0.37	0.22	0.61^a^
PUFA	19.59	3.63	19.86	3.67	0.53^a^	0.20	0.12	0.19	0.10	0.72^a^
Omega-3 FAs	1.80	0.63	1.82	0.61	0.89^a^	0.02	0.02	0.02	0.01	0.70^a^
Omega-6 FAs	17.79	3.54	18.04	3.51	0.52^a^	0.18	0.10	0.17	0.09	0.65^a^
	Relative mass proportion of polar lipids from all lipids									
	3.28	1.43	2.89	1.19	0.16^a^					

Data is presented as relative proportions (%) and quantities (mg/mL) and grouped based on milk cortisol levels. Statistically significant differences (p < 0.05) between low and high milk cortisol groups are highlighted with bold type. Abbreviations: STDEV standard deviation, SAFA saturated fatty acids, MUFA monounsaturated fatty acids, PUFA polyunsaturated fatty acids

^a^ Significant difference between the low and high cortisol groups, Mann–Whitney *U* test

^b^ Milk cortisol (as continuous variable) remained a statistically significant predictor after controlling for pre-pregnancy BMI and sampling time in linear regression analysis, see Additional file 1

^c^ Significant difference between the low and high cortisol groups, T-test (df = 98)

### Lipid and cortisol interactions

Among the TAG-rich lipids (Table 2), monounsaturated FAs (MUFAs) were most abundant in mass proportion (%) and they consisted mainly of oleic acid, (18:1*n* − 9). There was significantly more eicosenoic acid in the high cortisol group compared with the low cortisol group, but their overall prevalence was small. The content of saturated FAs (SAFAs) in the TAG-rich lipids was close to the level of MUFAs in mass proportions. Of the SAFA’s, palmitic acid, (16:0) was most abundant followed by stearic (18:0), myristic (14:0) and lauric (12:0) acids (Table 2). Higher cortisol level in breast milk was associated with lower 16:0 (*U =* 943, *p* = 0.03) but with higher proportion of lauric (12:0) (t = − 3.7, df = 98, *p* = 0.001) and myristic (14:0) acids (t = − 2.6, df = 98, *p* = 0.02) in the TAG-rich fraction of lipids. The linear regression analyses showed that after controlling for pre-pregnancy, BMI and sampling time, milk cortisol was shown to be significant for lauric acid (12:0) (mass %: β = 0.38, *p* = 0.000; mg/mL: β = 0.32, *p* = 0.003) and myristic acid (14:0) (mass %: β = 0.26, *p* = 0.01; mg/mL: β = 0.27, p = 0.01) in TAG-rich lipids [Additional file 1].

There were no differences in the mass proportions of total PUFAs or in the proportions of individual PUFAs between the cortisol groups (Table 2). The most abundant PUFA among the TAG-rich lipids was linoleic acid (18:2*n* − 6). The ratio of omega-6 FAs and omega-3 FAs in TAG-rich fraction did not differ between the groups.

PUFAs were more abundant in the PL-rich fraction than in the TAG-rich fraction (Table 3). The most abundant individual PUFAs in the PL-rich fraction were linoleic acid (18:2*n* − 6) followed by arachidonic acid (20:4*n* − 6). In the PL-rich fraction, higher cortisol was associated with higher myristic acid (14:0) (mass %; *U* = 887, *p* = 0.01) and 22:1*n* − 9 (mass %; *U* = 873, *p* = 0.009) (Table 3). After controlling for pre-pregnancy BMI and sampling time in linear regression, milk cortisol was shown to be significant for myristic acid (14:0) (mass %; β = 0.23, *p* = 0.03) and 22:1*n* − 9 (mass %; β = 0.23, p = 0.03) in PL-rich lipids [Additional file 1]. The ratio of omega-6 FAs and omega-3 FAs in the PL-rich fraction did not differ between the groups. Cortisol did not cause grouping in the PCA models with all of the measured lipids [Additional file 2].

### Separation of phospholipids in principal component analysis

In the principal component model with mass % data of PL-rich fraction, a group of 12 samples separated, but the separation was not associated with the milk cortisol [Additional file 3]. Based on the PCA correlation loadings, these samples were associated with a higher proportion of polyunsaturated fatty acids (especially those of belonging to the *n*–6 series) and stearic acid (18:0).

## Discussion

### Associations between cortisol concentrations and lipids in milk

Because glucocorticoids are known to take part in the regulation of lipid metabolism [6], this study investigated the associations between cortisol concentrations and lipid content and composition in human milk. Our findings were in agreement with an earlier report [25] that showed no correlation between milk glucocorticoids and total milk fat content. However, our study revealed that the influence of cortisol can be seen, when the composition of individual FAs in milk is investigated. Our study included milk samples with pre-identified low- and high cortisol concentrations. The composition of these milks differed in the abundance of lauric (12:0) and myristic (14:0) acids, with the high cortisol group having a higher prevalence and concentration of these FAs. This points to cortisol’s influence in the release of medium- and intermediate chain FAs from maternal body stores [6, 36]. Alternatively, these FAs may originate from endogenous de novo synthesis possibly indicating a high carbohydrate to lipid ratio in the mother’s diet [10, 37] or a high consumption of dairy fat where these FAs are abundant [20]. Lauric acid, as a medium-chain FA, provides rapidly available energy for the infant. We also found that lower milk cortisol level was associated with a higher proportion of palmitic acid (16:0), but this association was not significant when the model was adjusted for BMI and sampling time. The overall quantity of palmitic acid is important for the infant’s total energy intake, and its positioning in the glycerol backbone is of special importance to the infant. This is because palmitic acid released from the primary positions of the triacylglycerol backbone form insoluble calcium soaps, which are lost to feces and can harden the consistency of stools [37, 38]. Previously, minor monounsaturated FAs have been increased in the neural tissues of mice after dietary EPA and DHA feeding, and the authors concluded that their role in cognition should be investigated [39]. In this study, the proportion of eicosenoic acid in the breast milk was associated with higher cortisol levels, but the overall quantity or proportion in the milk was low in both groups investigated here, and the difference was not significant when adjusting the model with BMI and sampling time. However, our results point towards the need of further research also in the minor FAs such as eicosenoic and docosenoic acids.

### Association between cortisol and energy metabolism

Cortisol and obesity appear to be linked [40], as obesity is characterized by increased secretion of glucocorticoids [40, 41] and chronic exposure to glucocorticoids induces lipogenesis, resulting in increased fat storage and in particular, abdominal adiposity [7]. On the other hand, glucocorticoids in human milk reflect their circulating abundance in the maternal blood and saliva [42] and might play a role in the metabolic programming of the infant [43]. The fact that breastfed children exhibit 40% greater salivary cortisol than formula-fed children may indicate that the cortisol of milk is effectively absorbed into the infant’s circulatory system [44]. Previously, milk cortisone has been detected to be significantly higher in normal weight mothers compared to overweight and underweight women, but for cortisol no difference was seen [45]. In our study, low cortisol was associated with higher pre-pregnancy BMI. However, the average pre-pregnancy BMI of the mothers in our study was normal or very close to normal in both groups (25.12 ± 3.77 vs. 23.68 ± 4.47), and unfortunately, we had no information on BMI across pregnancy. After adjusting for pre-pregnancy BMI and milk sampling time, the association between milk cortisol and lauric and myristic acids in triacylglycerol-rich lipids, and myristic and docosenoic acid in phospholipid-rich lipids remained significant. Cortisol exposure through human milk may help to program metabolic functioning and influence the risk of childhood obesity via modulation of later glucocorticoid metabolism [46]. Infants exposed to higher milk cortisol levels at 3 months were less likely to exhibit BMI gains over the first 2 years of life, compared with infants exposed to lower milk cortisol, indicating that some level of cortisol exposure might be beneficial for developing healthy weight later [47]. This indicates that glucocorticoid exposure through human milk might have more complex effects on the development of obesity than systemic cortisol concentrations [48].

The effect of the composition of a single meal (mostly high protein vs. high carbohydrate) on cortisol has been previously studied, and the results vary from no effect [49, 50] to a higher response to a carbohydrate meal [51]. Some dietary supplements have been noted to affect steroid hormone homeostasis, as previously reviewed [52]. Examples of such supplements include the amino acid tryptophan [53] as well as phosphatidylserine and phosphatidic acid [54]. However, it is not clear whether these are relevant as part of a balanced diet. However, future studies investigating the effect of cortisol on human milk lipids would benefit from the assessment of the mother’s dietary profiles.

### Effect of polar membrane lipids in milk

The effect of polar membrane lipids is likely to extend beyond the content of long-chain PUFAs in them [26]. Previously, it has been shown that supplementation of the milk-fat globule membrane preparations to formula-fed infants has reduced the episodes of diarrhea [55] and has positively affected the cognitive score of the infants at the age of 12 [56]. In chronically stressed adult men, bovine milk based PLs have a delayed decline from peak levels in morning salivary cortisol and attenuated stress-induced memory impairments [57]. In our study, no correlation was found between the total amount of phospholipids in milk and milk cortisol. The conserved levels of phospholipids in human milk might be an indication of not only a certain conserved milk fat globule size but also of the importance of the globule membrane on the well-being and development of the infant.

### Effect of infant sex on milk composition

Previously, infant sex has been reported to affect milk energy density in macaque and human mothers [23, 58, 59]. In this study, no association between infant sex and the total amount of lipids or cortisol in the human milk was detected. The interaction between infant sex and milk cortisol has been shown to affect infant developmental outcomes as higher milk cortisol has been related to a higher infant fear reactivity in girls but not in boys [14]. Cortisol has also been linked with infant negative emotional reactivity, especially fear reactivity and sadness [33]. In addition, associations between postnatal glucocorticoid exposure and enhanced learning in rodents have been reported [60, 61] thus indicating the need for further research in the area of milk cortisol in relation to infant development.

### Strengths and limitations

This study had several strengths. The analyses of human milk lipids separately in the triacylglycerol-rich and phospholipid-rich fractions provided an indication of storage and membrane lipids, separately. Furthermore, the background data on the mother-infant dyads from the larger FinnBrain Birth Cohort Study utilized here were quite extensive. However, this study was limited in the number of hormones accounted for. While both cortisone and cortisol can be detected in milk, we chose to concentrate on milk cortisol, because it is more active than cortisone and has direct links with energy metabolism. Also, the present milk samples originated from an individual time point. The amount of cortisol in breast milk has been observed to be influenced by the time of the day of sampling and thus the use of frequent samples from all nursing occasions and calculations of an area under the cortisol curve would be feasible [42]. However, as our aim was to study the interrelationships between milk cortisol and milk lipids instead of overall cortisol exposure of the infants, and we used a regression model to adjust for sampling time that justified the use of an individual time point.

## Conclusions

Human breast milk represents one of the key early biological exposures for the developing child, including bioactive compounds like cortisol and fatty acids. Breast milk contains numerous bioactive molecules that take part at least in the protection against infections and inflammation, intestinal microbial colonization and contribute to organ, including neural, development [62]. The results of this study shed light in understanding the correlations between two bioactive components, namely the content and composition of lipids and cortisol. This study revealed small but significant associations between individual fatty acids of human milk and milk cortisol, indicating that cortisol might be one of the factors affecting the origin of the lipids in human milk. Such an association has not emerged, when only total fat content has been assessed. Thus milk cortisol might be one of the factors regulating the lipid composition in human milk but not necessarily its total lipid content. The principal component separation of phospholipid-rich lipids warrants further investigation of such minor but important components of human breast milk.

## Supplementary information

**Additional file 1: Supplementary Table 1**. Linear regression analysis.

**Additional file 2: Supplementary Figure 1**. Principal component analysis model with all mothers (*n* = 100) and all measured lipids.

**Additional file 3: Supplementary Figure 2**. Principal component analysis model of phospholipid fatty acids.

## Data Availability

The datasets analyzed during the current study are available from the corresponding author on a reasonable request.

## Abbreviations

BMI: Body mass index
FA: Fatty acid
MUFA: Monounsaturated fatty acid
TAG: Triacylglycerol
PL: Phospholipid
PUFA: Polyunsaturated fatty acid
SAFA: Saturated fatty acid
