# Supplementary material for: Interactions between cortisol and lipids in human milk
Source: Int Breastfeed J. 2020 Jul 20;15:66. doi: 10.1186/s13006-020-00307-7 (PMC7370511; doi:10.1186/s13006-020-00307-7)
Supplement: Supplementary file 1 — Additional file 1: Supplementary Table 1. Linear regression analysis. [file 13006_2020_307_MOESM1_ESM.pdf]

Supplementary Table 1. Linear regression analysis

**Dependent variable** [a]		**Model (R^2; adjusted R^2)**	**Constant / predictors**	**B (95% CI)** [b]	**SE B** [c]	**β** [d]	***p***
Lauric acid (12:0)	% in TAG	1 (0.02; −0.004)	Constant	4.71 (2.91, 6.52)	0.91		0.000
			Sampling time	0.001 (−0.002, 0.003)	0.001	0.04	0.70
			Pre-pregnancy BMI	−0.04 (−0.12, 0.03)	0.04	−0.13	0.21
		2 (0.14; 0.12)	Constant	2.82 (0.85, 4.78)	0.99		0.005
			Sampling time	0.002 (−0.001, 0.005)	0.001	0.16	0.12
			Pre-pregnancy BMI	−0.02 (−0.09, 0.04)	0.03	−0.07	0.50
			Milk cortisol	0.53 (0.25, 0.81)	0.14	0.38	0.000
	mg/mL in TAG	1 (0.000; −0.02)	Constant	1.36 (0.36, 2.36)	0.50		0.008
			Sampling time	-5.53×10^{-6} (−0.001, 0.001)	0.001	−0.001	0.99
			Pre-pregnancy BMI	−0.003 (−0.04, 0.04)	0.02	−0.01	0.90
		2 (0.09; 0.06)	Constant	0.49 (−0.61, 1.60)	0.56		0.89
			Sampling time	0.001 (−0.001, 0.002)	0.001	0.10	0.35
			Pre-pregnancy BMI	0.007 (−0.03, 0.05)	0.02	0.04	0.70
			Milk cortisol	0.25 (0.09, 0.40)	0.08	0.32	0.003
Myristic acid (14:0)	% in TAG	1 (0.006; −0.01)	Constant	6.30 (4.43, 8.16)	0.94		0.000
			Sampling time	0.000 (−0.002, 0.003)	0.001	0.03	0.79
			Pre-pregnancy BMI	−0.03 (−0.10, 0.04)	0.04	−0.08	0.45
		2 (0.07; 0.04)	Constant	4.97 (2.87, 7.07)	1.056		0.000
			Sampling time	0.001 (−0.001, 0.004)	0.001	0.11	0.30
			Pre-pregnancy BMI	−0.01 (−0.08, 0.06)	0.04	−0.31	0.72
			Milk cortisol	0.38 (0.08, 0.67)	0.15	0.26	0.01
	mg/mL in TAG	1 (0.003; −0.02)	Constant	1.65 (0.50, 2.80)	0.58		0.005
			Sampling time	0.000 (−0.001, 0.002)	0.001	0.04	0.71
			Pre-pregnancy BMI	0.007 (−0.04, 0.05)	0.022	0.03	0.77
		2 (0.07; 0.04)	Constant	0.82 (−0.47, 2.12)	0.653		0.21
			Sampling time	0.001 (−0.001, 0.003)	0.001	0.12	0.26
			Pre-pregnancy BMI	0.02 (−0.03, 0.06)	0.022	0.07	0.47
			Milk cortisol	0.23 (0.05, 0.42)	0.093	0.27	0.01
	% in PL	1 (0.03; 0.01)	Constant	5.53 (3.50, 7.56)	1.02		0.000
			Sampling time	−0.02 (−0.005, 0.001)	0.001	−0.17	0.11
			Pre-pregnancy BMI	−0.01 (−0.09, 0.07)	0.04	−0.04	0.73
		2 (0.08; 0.05)	Constant	4.24 (1.94, 6.55)	1.16		0.000
			Sampling time	−0.001 (−0.004, 0.002)	0.001	−0.09	0.37
			Pre-pregnancy BMI	0.001 (−0.08, 0.08)	0.04	0.002	0.98
			Milk cortisol	0.36 (0.04, 0.69)	0.17	0.23	0.03
Docosenoic acid (22:1*n*−9)	% in PL	1 (0.02; −0.001)	Constant	0. 22 (0.10, 0.35)	0.06		0.001
			Sampling time	-5.92×10^{-6} (0.000, 0.000)	0.000	−0.07	0.51
			Pre-pregnancy BMI	−0.003 (−0.008, 0.002)	0.002	−0.11	0.30
		2 (0.07; 0.04)	Constant	0.14 (0.000, 0.29)	0.07		0.05
			Sampling time	3.04×10^{-6} (0.000, 0.000)	0.000	0.003	0.97
			Pre-pregnancy BMI	−0.002 (−0.007, 0.003)	0.002	−0.07	0.47
			Milk cortisol	0.02 (0.002, 0.04)	0.01	0.23	0.03

The data represents the results of hierarchical multiple linear regression analysis for fatty acids and milk cortisol (as natural log-transformed), pre-pregnancy BMI and breast milk sampling time (minutes after 08:00 am).

[a] Abbreviations: TAG, triacylglycerol(-rich fraction); PL, phospholipid(-rich fraction)

[b] Unstandardized coefficients (B) with their 95% confidence interval (CI) in parentheses

[c] Standard error (SE) for unstandardized coefficient (B)

[d] Standardized coefficient beta (β)
