# Supplementary material for: Interactions between cortisol and lipids in human milk
Source: Int Breastfeed J. 2020 Jul 20;15:66. doi: 10.1186/s13006-020-00307-7 (PMC7370511; doi:10.1186/s13006-020-00307-7)
Supplement: Supplementary file 2 — Additional file 2: Supplementary Figure 1. Principal component analysis model with all mothers (n = 100) and all measured lipids. [file 13006_2020_307_MOESM2_ESM.pdf]

A

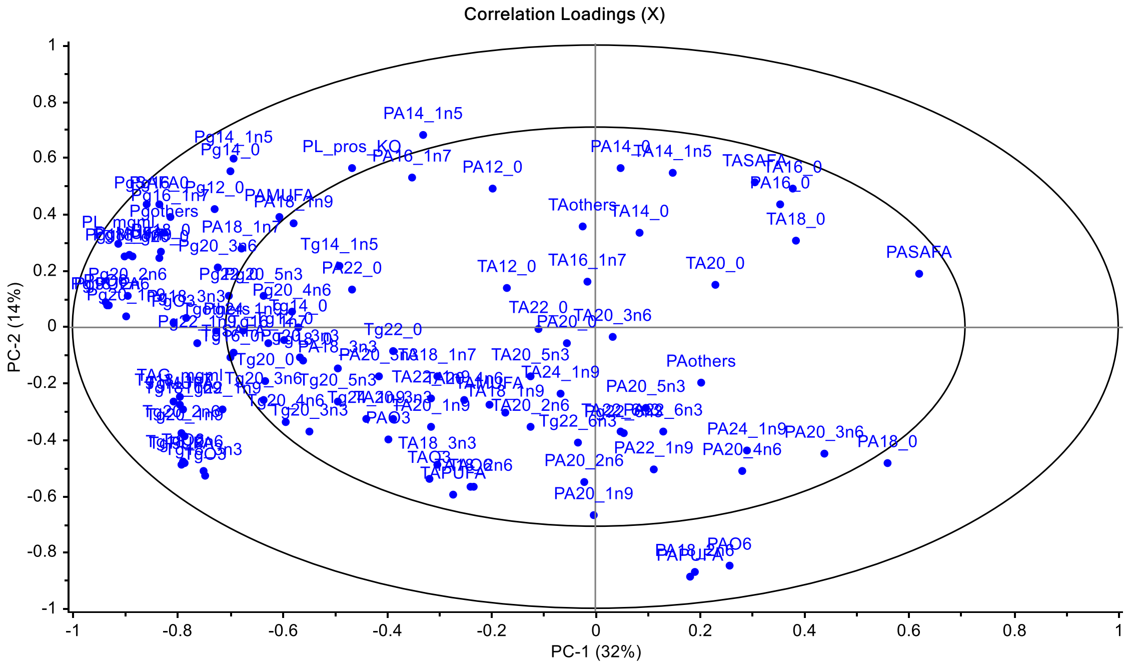

B

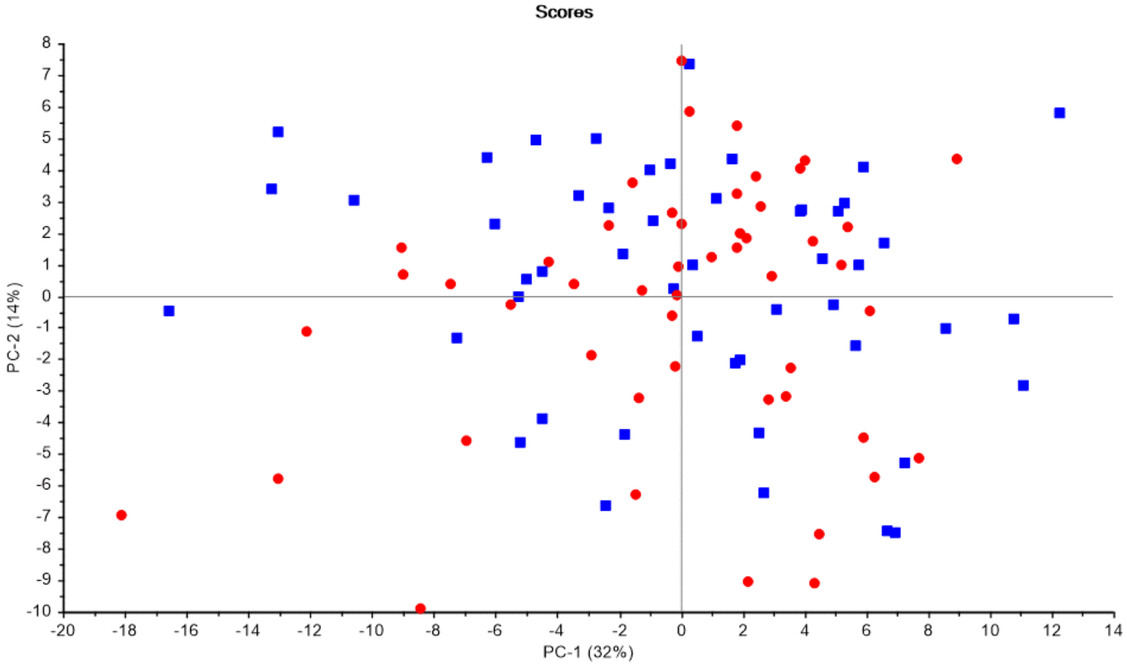

Supplementary Figure 1. Principal component analysis model with all mothers (*n* = 100) and all measured lipids. (**A**) Loadings plot. (**B**) Scores plot: Low cortisol group marked with blue square; High cortisol group marked with red circle. Label abbreviations for variables: PA, relative abundance of fatty acid in phospholipids; Pg, mg/mL fatty acid in phospholipids; TA, relative abundance of fatty acid peak in triacylglycerols; Tg, mg/mL fatty acid in triacylglycerols.
