# Supplementary material for: Interactions between cortisol and lipids in human milk
Source: Int Breastfeed J. 2020 Jul 20;15:66. doi: 10.1186/s13006-020-00307-7 (PMC7370511; doi:10.1186/s13006-020-00307-7)
Supplement: Supplementary file 3 — Additional file 3: Supplementary Figure 2. Principal component analysis model of phospholipid fatty acids. [file 13006_2020_307_MOESM3_ESM.pdf]

A

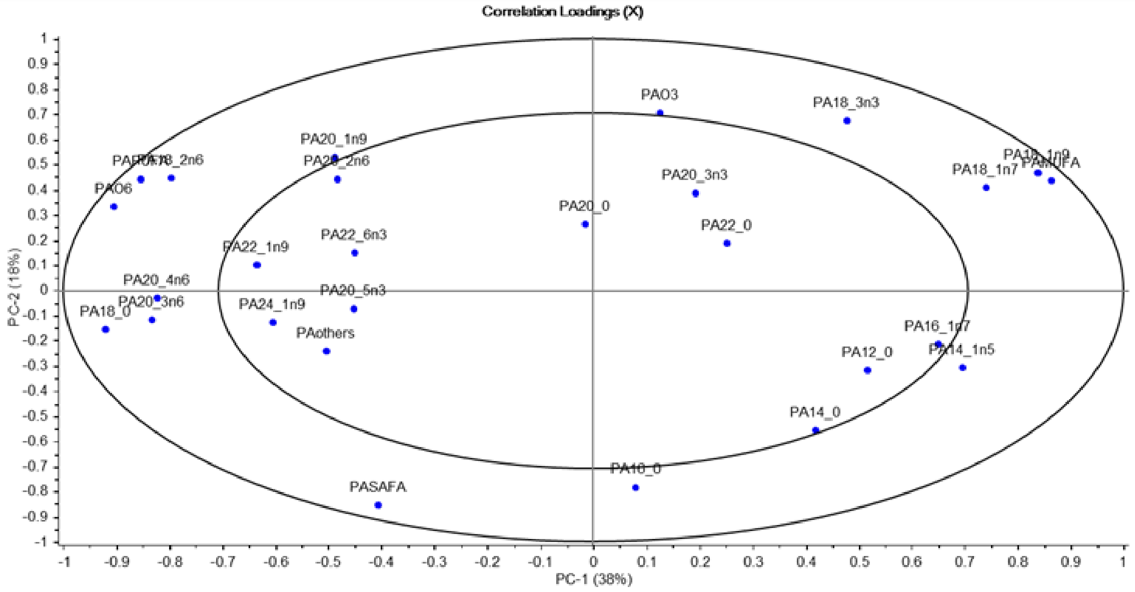

B

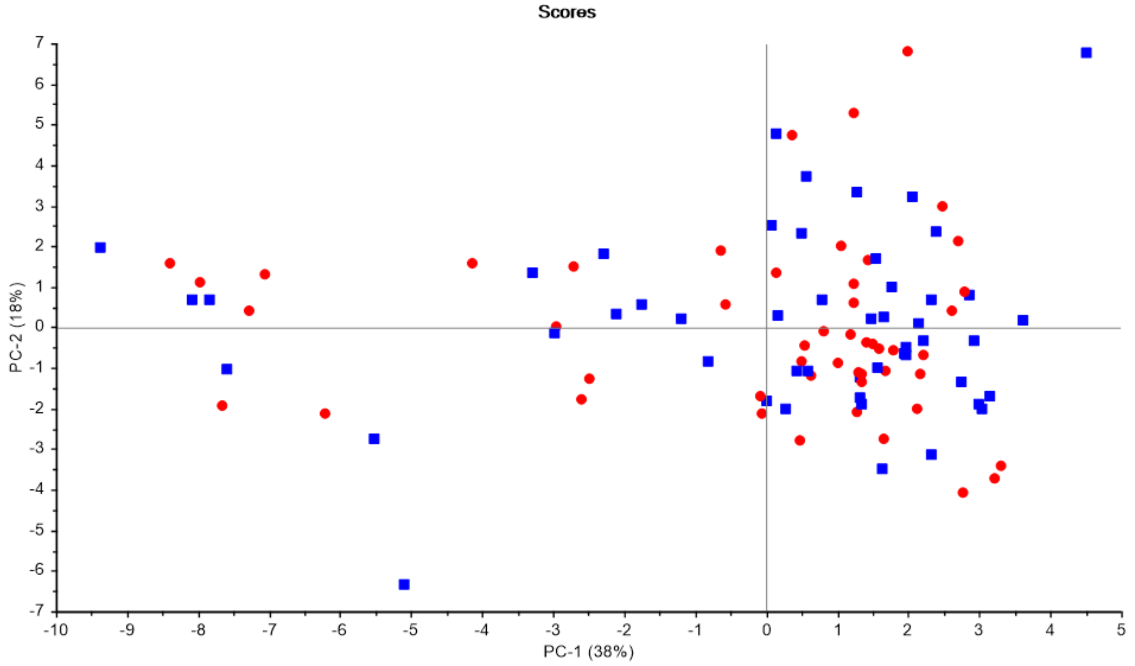

Supplementary Figure 2. Principal component analysis model of phospholipid fatty acids. The data is represents the relative abundancies of phospholipid fatty acids (PA). (**A**) Loadings plot. (**B**) Scores plot: Low milk cortisol (red circle; *n* = 50); High milk cortisol (blue square; *n* = 50). A separation of 12 samples is seen from the model. The separation was not based on milk cortisol.
